# Role of biomarkers in the management of antibiotic therapy: an expert panel review II: clinical use of biomarkers for initiation or discontinuation of antibiotic therapy

**DOI:** 10.1186/2110-5820-3-21

**Published:** 2013-07-08

**Authors:** Jean-Pierre Quenot, Charles-Edouard Luyt, Nicolas Roche, Martin Chalumeau, Pierre-Emmanuel Charles, Yann-Eric Claessens, Sigismond Lasocki, Jean-Pierre Bedos, Yves Péan, François Philippart, Stéphanie Ruiz, Christele Gras-Leguen, Anne-Marie Dupuy, Jérôme Pugin, Jean-Paul Stahl, Benoit Misset, Rémy Gauzit, Christian Brun-Buisson

**Affiliations:** 1 Service de réanimation médicale, CHU Dijon & Université de Bourgogne, 14 rue Paul Gaffarel, 21970 Dijon, France; 2 Centre d’investigation clinique, INSERM CIE 1, 7 Boulevard Jeanne d’Arc, 21079 Dijon, France; 3 Service de Réanimation Médicale, Institut de Cardiologie, Groupe Hospitalier Pitié-Salpêtrière, AP-HP, Université Pierre et Marie Curie - Paris VI, 47-83 boulevard de l'Hôpital, Cedex 13, 75651 Paris, France; 4 Service de Pneumologie et Soins Intensifs Respiratoires, Hôpitaux Universitaires Paris Centre, AP-HP & Université Paris-Descartes, 27 rue du fbg St Jacques, 75679 Paris, France; 5 Service de Pédiatrie Générale, CHU Necker-Enfants Malades, AP-HP, Université Paris Descartes, 149 rue de Sèvres, 75743 Paris, France; 6 Inserm U953, Paris, France; 7 Laboratoire Interactions Muqueuses Agents Pathogènes, EA562, UFR Médecine, Université de Bourgogne, 7 Bd Jeanne d'Arc, 21000 Dijon, France; 8 Département d’Urgences Médicales, Centre Hospitalier Princesse Grace, 1 ave Pasteur, BP 489, Principauté de Monaco, 98012 Monaco; 9 Pôle d'Anesthésie Réanimation, CHU d’Angers, 4 rue Larrey, 49933 Cedex 9 Angers, France; 10 Service de réanimation, Centre hospitalier de Versailles, 177, rue de Versailles, 78150, Le Chesnay, France; 11 Laboratoire de Microbiologie, Institut Mutualiste Montsouris, 42, Bld Jourdan, 75014 Paris, France; 12 Service de réanimation polyvalente, Groupe hospitalier Paris Saint Joseph, 185 rue Raymond Losserand, 75014 Paris, France; 13 Pôle d'Anesthésie-Réanimation, Hôpital de Rangueil, CHU de Toulouse, 1, Ave Pr Jean Poulhès, TSA 50032, Cedex 9, 31059 Toulouse, France; 14 Clinique Médicale et Service d'Urgences Pédiatriques, Hôpital Mère-Enfant, CHU Nantes, 38 boulevard Jean-Monnet, 44093 Nantes cedex 1, France; 15 Département de Biochimie, Hopital Lapeyronie, CHU Montpellier, 371, avenue du doyen Gaston Giraud, Cédex 5, 34295 Montpellier, France; 16 Intensive Care - SIRS Unit, University Hospitals of Geneva, 4 rue Gabrielle Perret-Gentil, 1211 Geneva 14, Switzerland; 17 Service de maladies infectieuses et tropicales, Université 1 de Grenoble, CHU de Grenoble, BP 217, Boulevard de la Chantourne, 38043 Grenoble, France; 18 Centre de Recherche Clinique, Groupe hospitalier Paris Saint Joseph, Université Paris Descartes, 185 rue Raymond Losserand, 75014 Paris, France; 19 Unité de réanimation, CHU Hôtel Dieu, AP-HP, Place du Parvis-de-Notre-Dame, 75004 Paris, France; 20 Service de Réanimation médicale, Hôpitaux Universitaires Henri Mondor, AP-HP & Université Paris-Est, 51, av de Lattre de Tassigny, 94000 Créteil, France; 21 Inserm U957, Institut Pasteur, Paris, France

**Keywords:** Infection, Sepsis, Emergency medicine, Biomarkers, Procalcitonin, C-reactive protein, Pancreatitis, Meningitis, Pneumonia

## Abstract

Biomarker-guided initiation of antibiotic therapy has been studied in four conditions: acute pancreatitis, lower respiratory tract infection (LRTI), meningitis, and sepsis in the ICU. In pancreatitis with suspected infected necrosis, initiating antibiotics best relies on fine-needle aspiration and demonstration of infected material. We suggest that PCT be measured to help predict infection; however, available data are insufficient to decide on initiating antibiotics based on PCT levels. In adult patients suspected of community-acquired LRTI, we suggest withholding antibiotic therapy when the serum PCT level is low (<0.25 ng/mL); in patients having nosocomial LRTI, data are insufficient to recommend initiating therapy based on a single PCT level or even repeated measurements. For children with suspected bacterial meningitis, we recommend using a decision rule as an aid to therapeutic decisions, such as the Bacterial Meningitis Score or the Meningitest®; a single PCT level ≥0.5 ng/mL also may be used, but false-negatives may occur. In adults with suspected bacterial meningitis, we suggest integrating serum PCT measurements in a clinical decision rule to help distinguish between viral and bacterial meningitis, using a 0.5 ng/mL threshold. For ICU patients suspected of community-acquired infection, we do not recommend using a threshold serum PCT value to help the decision to initiate antibiotic therapy; data are insufficient to recommend using PCT serum kinetics for the decision to initiate antibiotic therapy in patients suspected of ICU-acquired infection. In children, CRP can probably be used to help discontinue therapy, although the evidence is limited. In adults, antibiotic discontinuation can be based on an algorithm using repeated PCT measurements. In non-immunocompromised out- or in- patients treated for RTI, antibiotics can be discontinued if the PCT level at day 3 is < 0.25 ng/mL or has decreased by >80-90%, whether or not microbiological documentation has been obtained. For ICU patients who have nonbacteremic sepsis from a known site of infection, antibiotics can be stopped if the PCT level at day 3 is < 0.5 ng/mL or has decreased by >80% relative to the highest level recorded, irrespective of the severity of the infectious episode; in bacteremic patients, a minimal duration of therapy of 5 days is recommended.

## Biomarkers and initiation of antibiotic therapy

According to the preset selection criteria (see part I), the panel reviewed four conditions in which the potential clinical role of biomarkers has been studied: acute pancreatitis, respiratory tract infections, meningitis, and sepsis in the ICU.

### Acute pancreatitis in adults

The clinical presentation and severity of patients having acute pancreatitis varies considerably, from a mild abdominal discomfort to multiple organ failure and death. The potential role of biomarkers in this condition should thus be twofold: 1) a prognostic value, to help define the most appropriate therapeutic approach, by predicting the severity of the disease and accurately select those patients needing close monitoring in the ICU; and 2) a diagnostic value, to help identify those patients having infected pancreatic necrosis, who might need drainage or surgery. Mofidi et al. [1] have recently reviewed the potential role of PCT in answering these two questions, by analysing 12 observational studies [2-9] totalling 956 patients. The threshold PCT value used in these studies to predict the severity of pancreatitis varied from 0.25 to 1.8 mg/L, with an associated combined sensitivity of 0.72 (0.65-0.78), a specificity of 0.86 (0.83-0.89), and an area under the receiver operating curve (AUROC) of 0.87. In the seven studies (n = 264 patients) examining the value of PCT for predicting the presence of infected necrosis [2-5,7-9], the threshold value varied across studies between 0.48 and 3.5 mg/L, with an associated sensitivity of 0.8 (0.71-0.88), a specificity of 0.91 (0.87-0.94), and an AUROC of 0.91 (Table 1). In these seven studies, PCT levels were confronted to microbiological results obtained from fine-needle biopsy and culture of intra-abdominal collections, taken as the “gold standard.”

**Table 1:** Use of biomarkers for the diagnosis of infected necrosis secondary to acute pancreatitis

**Marker**	**Study**	**Study design**	**Nb of patients, *n***	**Level of evidence**	**Biomarker tested and groups compared**	**Main results**
	***1***^***st ***^***author, [Ref]***					
**PCT/CRP**	Rau B, [2]	Observational	61	Low	Comparison of PCT and CRP levels between 3 groups:	AUROC for the diagnosis of infected necrosis:
					Oedematous pancreatitis (n = 22)	PCT (>1.8 mg/L) = 0.95 (Se: 95%, Sp: 88%),
					Sterile necrosis (n = 18)	CRP (>300 mg/L) = 0.86 (Se:86%, Sp: 75%); *p* < 0.02
					Infected necrosis (n = 21) according to imaging/surgery/microbiological data	
**PCT/CRP/GCSF**	Muller CA, [3]	Observational	64	Low	Comparison of PCT, G-CSF, and CRP between patients having oedematous pancreatitis (n = 29)	AUROC for diagnosing infected necrosis:
						CRP (>250) = 0.79 (Se: 83, Sp: 70%), PCT (0.45) = 0.77 (Se: 92%, Sp: 65%), AUC G-CSF (101) = 0.72 (Se: 92%, Sp: 48%)
					Noninfected necrosis (n = 23)	
					Infected necrosis (n = 12) according to imaging/surgery/microbiological data	
**PCT/CRP/IL8**	Rau B, [4]	Observational	50	Low	Comparison of PCT, IL8, and CRP levels between patients with:	AUROC for diagnosing infected necrosis:
					Oedematous pancreatitis (n = 18)	CRP (>300) = 0.84 (Se: 83, Sp: 78%),
					Non-infected necrosis (n = 14)	PCT (>1.8) = 0.97 (Se: 94%, Sp: 90%)
					Infected necrosis (n = 18) according to imaging/surgery/microbiological data	IL-8 (112) = 0.78 (Se: 72%, Sp: 75%)
**PCT/CRP/IL6/TNF**	Riche F, [5]	Observational	48	Low	Comparison of PCT, IL-6, TNF-α, and CRP between patients having	AUROC for diagnosing infected necrosis:
					- Noninfected necrosis (n = 33)	CRP = 0.76,
					- Infected necrosis (n = 15), according to imaging/surgery/microbiological data	PCT = 0.78,
						IL 6 = 0.77,
						TNF α = 0.5
**PCT**	Purkayastha S, [6]	Literature review (5 studies)	206	Low	Assessing the value of PCT for diagnosing infected pancreatic necrosis	Threshold values for PCT vary from 0.48 to 2;
						Sensitivity: 0.73 to 0.94
						Specificity: 0.65 to 1
**PCT/IL6/TNF/sTREM1**	Lu Z, [7]	Observational	30	Low	Comparison of PCT, IL-6, TNF-α, and sTREM-1 levels in serum and drainage fluid between patients having:	Biomarker levels in drainage fluid: No difference between the two groups for CRP, TNF-α, and IL-6 levels
					- Noninfected necrosis (n = 12), or	- sTREM1 (287), AUC = 0.97 (Se = 94, Sp = 92)
					- Infected necrosis (n = 18), according to imaging/surgery/microbiological data	- PCT (2.1): AUC = 0.9 (Se = 86, Sp = 91).
						Lower AUCs for serum levels:
						PCT: 0.79; sTREM1: 0.73
**PCT**	Olah A, [8]	Observational	24	Low	Comparison of PCT levels in patients having	Serum PCT level >0.5 predicts infected necrosis with Se = 75% and Sp = 83%.
					- Noninfected necrosis (n = 12)	Fine-needle aspiration predicts infection with Se = 92% and Sp = 100%.
					- Infected necrosis (n = 12)	
					According to results of fine-needle aspiration and culture and surgery	
**PCT/IL6/sICAM1**	Mandi Y, [9]	Observational	30	Low	Comparison of PCT, IL-6, and sICAM-1 between patients with	Only PCT (threshold >1 mg/L) allowed to distinguish patients with or without infected necrosis (Se = 90%; Sp = 100%).
					Noninfected necrosis (n = 10)	
					Infected necrosis (n = 10), according to results of biopsy and culture.	
**PCT**	Mofidi R, [1]	Literature review (7 studies)	264	Low	Assessment of PCT serum levels for the diagnosis of infected pancreatic necrosis	Threshold values vary from 0.48 to 3.5 mg/L, with a sensitivity of 0.63 to 0.92 and specificity of 0.71 to 0.97.
**Summary table: infected necrosis in acute pancreatitis**						
Number of studies, n	Total number of patients, n	Highest level of evidence	Directness*	Consistency of results**	Overall strength of evidence	
7	264^a^	Low	Yes		Yes	Moderate

^a^Number of patients included in diagnostic studies of infected pancreatic necrosis.

*Directness: studies provide evidence of a direct association between a treatment or a given risk factor and a judgment criterion.

**Consistency: results from studies of similar level of evidence are not contradictory.

Other less commonly measured biomarkers (IL-6, IL-8, sTREM-1, TNF-α) also have been compared to PCT for their ability to help answer the two questions above. These biomarkers provided AUROC comparable to those of PCT, both in terms of prognostic value and of diagnosis of infected necrosis [2,4-6,9,10]. Conversely, CRP levels appear less discriminatory for the prediction of infected necrosis [2]. No study has evaluated the value of repeated PCT measurements to predict infection, and no study has evaluated the impact on patients’ outcome of the initiation of antibiotic therapy guided by a biomarker level in patients suspected of infected necrosis.

In summary, we suggest that PCT be measured to help predict infection in patients suspected of infected necrosis during acute pancreatitis; it is however difficult from the available literature to define a precise threshold value (0.5-1.0 mg/L). A PCT value above the threshold might reinforce the clinician’s judgment that a fine-needle aspiration and culture is needed to confirm infection, while a value below this threshold might help deferring this intervention and proceed with watchful waiting. There is insufficient data to recommend initiating antibiotic therapy based on biomarker levels: this decision is based on a careful repeated evaluation of the patient and on the results of fine-needle aspiration material, which currently remains the cornerstone for the decision to initiate or maintain antibiotic therapy.

### Lower respiratory tract infection in adults

Antibiotics often are prescribed in excess to patients having a clinical syndrome of community-acquired lower respiratory tract infection (LRTI). Despite the usually viral aetiology of their illness, an estimated 75% of patients with acute bronchitis receive antibiotics [11]; indeed, clinical presentation does not allow the distinction between bacterial and viral infection, which encourages physicians to err on the “safe side” and prescribe antibiotics. Communication campaigns inciting primary physicians to limit unnecessary prescriptions for LRTI have a moderate impact, which is difficult to maintain over time [12,13]. In this context, the addition of biomarker measurements to the clinical evaluation of such patients may have two main potential effects: improve the diagnostic accuracy, and reassure the patient and the physician that antibiotic therapy is unnecessary.

An abundant literature is available on PCT-guided initiation of antibiotic therapy in patients suspected clinically of having LRTI, providing a high-level of evidence. To date, 11 randomised, controlled studies using a similar approach have been published and provide consistent results [14-25]. All these studies have used a similar algorithm [26-29] to help decide on the initiation and continuation of antibiotic therapy, with a lower PCT threshold of <0.25 ng/mL to encourage physicians to withhold antibiotic prescription. The absolute risk reduction of antibiotic administration varies between 11% and 72% across these studies compared with “usual care” based on local recommendations and physicians’ judgment and preferences (Table 2). In one study, however, antibiotic prescriptions increased by 6% with PCT-guided therapy [19]. It also should be noted that the 0.25 ng/mL threshold may be less reliable in the elderly, where an 8% false-positive rate has been reported [30].

**Table 2:** Role of biomarkers in the initiation of antibiotic therapy for lower respiratory tract infection

**Biomarker**	**Study (ref)**	**Study design**	**Nb patients, *n (setting)***	**Level of evidence**	**End-point**	**Main results, absolute risk reduction (ARR) or odds ratio (OR; 95% CI)**
	***1***^***st ***^***author, [Ref]***					
**PCT**	Stolz D, [20]	Single-centre, randomised, controlled open study	208	High	Antibiotic exposure and rate of initiation of antibiotic therapy, based on PCT level > 0.25 μg/L	ARR = 32% (40% vs. 72%) of antibiotic prescriptions in the PCT-guided group.
			(AECB)			
						Ab exposure OR = 0.56 [0.43-0.73]
**PCT**	Schuetz P, [25]	Multicentre, open RCT	1359	High	Antibiotic exposure	ARR = 12% (75.4% vs. 87.7%) in PCT group, Overall antibiotic exposure = - 35% (5.7 vs. 8.7 days).
		Noninferiority study	(ED)		Based on a PCT level > 0.25 μg/L for initiating prescription.	
**PCT**	Christ-Crain M, [18]	Single-centre open RCT	302	High	Antibiotic initiation rate	ARR = 14% (85% vs. 99%) in initial antibiotic prescription in PCT group
			(ED, ward)		Antibiotic exposure	
					Based on a PCT level > 0.25 μg/L to initiate therapy	Overall ab exposure: OR = 0.52 [0.48–0.55]
**PCT**	Kristoffersen KB, [19]	Single-centre, open, RCT	210	High	Antibiotic prescription rate, based on a PCT level > 0.25 μg/L to initiate therapy in PCT group	3% increase in antibiotic prescription (88% vs. 85%) in the PCT group
			(ED, ward)			
**PCT**	Long W, [23]	Single-centre, open RCT	127	High	Antibiotic prescription rate, based on a PCT level > 0.25 μg/L in the PCT group	ARR = 11% of antibiotic prescriptions in the PCT group
			(ED)			
**PCT**	Long W, [22]	Single-centre, open RCT	156	High	Antibiotic prescription rate, based on a PCT level > 0.25 μg/L in the PCT group	ARR = 13% of antibiotic prescriptions in the PCT group
			(ED)			
**PCT**	Burkhardt O, [16]	Single-centre, open RCT, noninferiority	550	High	Antibiotic prescription rate, based on a PCT level > 0.25 μg/L in the PCT group	ARR = 15% (21.5% vs. 36.7%) for antibiotic prescription rate in the PCT group
			(PC)			
**PCT**	Briel M, [15]	Multicentre, open RCT, noninferiority	458	High	Antibiotic prescription rate, based on a PCT level > 0.25 μg/L in the PCT group	ARR = 72% [95% CI 66-78] for antibiotic prescription rate in the PCT group
			(PC)			
**PCT**	Schuetz P, [30]	Meta-analysis of 14 RCTs	3 119	High		Risk reduction of initial antibiotic therapy: OR = 0.24 (95% CI, 0.2-0.29)
						Overall antibiotic exposure:
						OR = 0.1 (95% CI = 0.07-0.14), without difference in mortality rates
**PCT**	Van der Meer V, [28]	Literature review on the use of CRP (13 studies)	13	High	Prediction of LRTI	Bacterial LRTI predicted with a sensitivity varying from 8% to 99% and a specificity varying from 27% to 95%
**PCT**	Schuetz P, [29]	Review of 8 RCTs using an PCT-based algorithm for the initiation of antibiotic therapy	3 457	High	Antibiotic prescription rate	ARR varying from 6% to 72%
**CRP**	Cals JW, [24]	Multicentre, open cluster-RCT, testing a CRP-based algorithm	431	High	Antibiotic prescription rate and antibiotic exposure, based on a CRP value < 20 : no antibiotic; CRP >100 : atb recommended, and 20<CRP<99 : reassess for possible therapy	ARR = 22% (31% vs. 53%) of initial antibiotic prescriptions in the CRP group
						Overall antibiotic exposure: - 13% (45% vs. 58%)
**PCT**	Christ-Crain M, [17]	Multicentre, open, cluster-RCT	243	High	Antibiotic prescription rate, based on a PCT level > 0.25 μg/L in the PCT group	ARR = 39% for antibiotic prescription rate in the PCT group
			(ED)			
**Summary of evidence table: Lower respiratory tract infection**						
**Number of studies, n**	**Total number of patients, n**	**Highest level of evidence**	Directness*	Consistency**	**Overall strength of evidence**	
12	4 412	High	Yes	Yes	Strong	

*Directness: studies provide evidence of a direct association between a treatment or a given risk factor and a judgment criterion.

**Consistency: results from studies of similar level of evidence are not contradictory.

Among the 14 studies of PCT-guided therapy for LRTI reviewed by the Cochrane Collaboration [31,32], only 3 enrolled patients with a nosocomial infection (hospital-acquired or ventilator-associated) [33-35], 2 of which evaluated the impact of PCT-guided therapy on the initiation of treatment [33,34]. However, nosocomial acquisition of infection is identifiable only in the study by Bouadma et al. [33], and only 5% (n = 141) of all patients enrolled fulfilled this criteria; nearly all patients in this subgroup were administered antibiotics (99% in the PCT-guided therapy group and 100% in controls). Repeated measurements might be helpful for initiating antibiotics in this subgroup; however, the few data available on a limited number of patients (n = 89 patients) [36] do not allow making a recommendation in this regard.

In summary, we suggest withholding antibiotic therapy in adult patients suspected of community-acquired LRTI and having a serum PCT level <0.25 ng/mL; if clinical suspicion is high, it is however recommended to repeat the PCT measurement at a 6-h interval and reassess the therapeutic approach, accounting for new clinical findings. In patients having nosocomial LRTI, data are insufficient to recommend tailoring the therapeutic approach based on a single PCT level or even repeated measurements.

### Meningitis

#### *Childhood meningitis*

Most acute meningitis in children is of viral aetiology and evolves favourably [37,38]. Despite their relatively low prevalence, acute bacterial meningitis are severe infections, often resulting in debilitating sequels or even death [39]; thus, antibiotic therapy is recommended in children presenting with acute meningitis, at least until cerebrospinal fluid (CSF) cultures are available, i.e., within the first 48–72 h [40]. The risk-benefit ratio and costs associated with this prudent approach is likely unfavourable, because it involves numerous unnecessary hospitalisations, increased costs, and side effects of treatments, including selection of resistant organisms [41]. Biomarkers might help to reduce these unwanted effects [42,43]. Ideally, a good biomarker would have 100% sensitivity for the diagnosis of bacterial meningitis, together with an acceptable specificity [38]; however, when used alone, available biomarkers (PCT, CRP, IFN-Υ, etc.) have sensitivities and specificities that do not appear high enough to base a therapeutic decision on their results given the risks incurred in case of a false-negative test [44,45].

To overcome this problem, several groups have proposed decision rules combining clinical criteria and biomarker results [46-50]. The Bacterial Meningitis Score (BMS) [46] has been reported to have 100% sensitivity and 67% specificity for the detection of bacterial meningitis and is easily applicable at the bedside. This decision rule encourages ambulatory treatment of children having meningitis (i.e., a CSF leucocytes count ≥7/mm^3^) if none of the following five criteria is present: seizures, blood polymorphonuclear (PMN) cells count ≥10,000/mm^3^, direct examination of CSF positive, CSF protein level ≥0.8 g/L, or CSF PMN ≥1000/mm^3^. The BMS has undergone external validation on the large database of the French national registry for childhood bacterial meningitis (ACTIV-GPIP) [51]. Of 889 children with confirmed bacterial meningitis, 884 were correctly identified by the BMS rule (sensitivity = 99.6%; 95% confidence interval (CI) 98.9-99.8) with a specificity >60%. Thus, despite these near-perfect results, a few patients with bacterial meningitis (n = 5) were not detected by the BMS [52]; that the BMS can have a few false-negatives also was confirmed by a recent meta-analysis [53]. The Meningitest**®** (European patent EP1977244) has been subsequently proposed to refine the BMS and avoid these false-negatives, by omitting some variables of poor discriminatory power and introducing the serum PCT level [45,54]. The Meningitest**®** rule suggests initiating antibiotic therapy if at least one of the following criteria is present: seizures, toxic appearance, purpura, PCT level ≥0.5 ng/mL, positive CSF Gram stain, or CSF protein level ≥0.5 g/L. External validation of the Meningitest**®** has been performed on an European database of 198 patients (including 96 with bacterial meningitis), where its sensitivity and specificity were respectively of 100% (95% CI 96–100) and 36% (95% CI 27–46) for the diagnosis of bacterial meningitis, whereas corresponding values for the BMS were 100% (95% CI 96–100) and 52% (95% CI 42–62) [55]. A single serum PCT level ≥0.5 ng/mL has similar sensitivity and specificity as the BMS, whereas combining CRP with CSF protein levels provided lower performances.

In summary, for children with suspected bacterial meningitis, we recommend using a decision rule as an aid to triage decisions and antibiotic prescribing, such as the BMS (more specific, but with a few false-negatives) or the Meningitest**®** (less specific, but no false-negative described to date). A single PCT level ≥0.5 ng/mL also may be used, but false-negatives may occur.

#### *Adult meningitis*

The potential role of biomarkers in the management of meningitis has been much less studied in adults than in children. Similarly to children, the use of a clinical decision rule to distinguish between viral and bacterial meningitis is recommended in adults [56]. For example, the French 2008 consensus conference on meningitis recommended using one of three decision rules: the rule developed by Hoen et al. [57], the BMS, or the Meningitest® [56]. It should be noted that the former rule has insufficient sensitivity in children (94%), with a risk of false-negatives [45].

Knudsen et al. have examined the impact of various biomarkers in the diagnostic workup of 55 adult patients with meningitis [58]. These authors found an AUROC of 0.91, 0.87, and 0.72 for CRP, PCT, and sCD 163, respectively, and concluded that CRP and PCT levels could be useful when combined with results of CSF examination to help diagnose bacterial meningitis. One recent study [59] included 151 patients admitted to an adult emergency department with suspected of bacterial meningitis and a negative direct examination of CSF, to assess the diagnostic value of CRP, PCT, and CSF leucocytes count. The AUROC of PCT and CRP were 0.98 (95% CI, 0.83-1.0) and 0.81 (95% CI, 0.58-0.92), respectively; however, the small number of patients with confirmed bacterial meningitis (n = 18) limits the inferences from this study. Of note, the CSF leucocytes count appeared to have little discriminatory value (AUC = 0.59) in that study. In another study of 30 patients (including 16 having bacterial meningitis), Schwarz et al. [60] found that PCT had a sensitivity of 69% and a specificity of 100% for diagnosing bacterial meningitis. In another larger prospective study that included 112 adult patients admitted to the hospital for meningitis (90 viral and 22 bacterial), Viallon et al. [61] found that a serum PCT value >0.93 ng/ml was 100% sensitive for the diagnosis of bacterial meningitis; conversely, a CSF lactate level <3.2 mmoles/L had a 100% NPV (Table 3). Low CRP levels have high NPV, but have not been shown to contribute markedly to the diagnostic approach [62]. The 2008 French consensus conference on management of acute bacterial meningitis [56] concluded that these biomarkers could be helpful for diagnosing bacterial meningitis in adults, pointing out that a threshold value for serum PCT of 0.5 ng/mL had a high sensitivity (99%; 95% CI, 97–100) and specificity (83%; 95% CI, 76–90), and that bacterial meningitis could be considered very unlikely when PCT was <0.5 ng/mL or CSF lactate was below 3 mmoles/L.

**Table 3:** Studies of biomarkers in the diagnosis of bacterial meningitis (BM) and its distinction from viral meningitis (VM)

**Marker**	**Study *1st author, [ref]***	**Study design**	**Number of patients, *n***	**Level of evidence**	**End-point**	**Main result**
						**Absolute risk reduction (RR)**
**PCT**	Gendrel D, [42]	Single-centre observational study	59 children (18 BM, 41 VM)	Very low	Comparison of PCT and CRP level in patients with bacterial or viral meningitis	A serum PCT level >0.5 μg/L is associated with bacterial meningitis (Se = 94%; Sp = 100%).
						Large overlap for CRP values
**PCT/CRP/INF-ϒ**	Marc E, [43]	Single-centre observational study	58 viral	Very low	Antibiotic initiation and hospital days, based on a serum PCT < 0.5 to not initiate or stop antibiotics. If PCT >0.5, antibiotics stopped if negative cultures and/or INF or PCR (+) in CSF	41 patients did not receive antibiotics; antibiotics stopped in 15/17 pts treated by day 1 or 2, because of a PCT < 0.5.
			(enterovirus outbreak)			
			Children (2 mo – 14 yr)			
						Hospital days reduced to 2 days.
**PCT/CRP/sCD 163**	Knudsen T, [58]	Single-centre observational study (ID department)	55 adult patients suspected of BM	Very low	Comparison of PCT, CRP and sCD163 levels in patients with bacterial or viral meningitis, or other infection	Diagnostic value of CRP (AUC = 0.91) and PCT (AUC = 0.87) superior (*p* < 0.02 and *p* < 0.06) to sCD163 (AUC = 0.72);
						sCD163 most specific for systemic bacterial infection (Sp = 0.91).
**PCT/CRP**	Viallon A [61]	Single-centre observational study	254 adults (183 VM, 97 BM)	Low	Predictive value of serum PCT and CRP for the diagnosis of BM	AUROC PCT = 0.86; threshold 0.28 μg/mL (Se = 0.97; Sp = 1, VPP = 0.97, VPN = 1)
						AUROC CRP = 0.92; threshold 37 mg/L (Se = 0.86, Sp = 0.84, VPP = 0.46, VPN = 0.97)
**CRP**	Gerdes L, [62]	Meta analysis of 10 studies	Children and adults	low	Predictive value of serum CRP for bacterial meningitis	Threshold value for CRP varies across studies from 19 to 100 mg/l.
						Se varies from 92% to 94% and NPV is >97%.
**Summary table**						
	**Total number of patients**	**Highest level of evidence**	Directness*	Consistency of results**	**Overall strength of evidence**	**Number of studies**
	371	Low	Yes	Yes	Low	3 (PCT)

*Directness: studies provide evidence of a direct association between a treatment or a given risk factor and a judgment criterion.

**Consistency: results from studies of similar level of evidence are not contradictory.

In summary, we suggest integrating serum PCT measurements in a clinical decision rule for meningitis in adults to help distinguish between viral and bacterial meningitis, using a threshold of 0.5 ng/mL.

### Adult intensive care patients

Most controlled studies performed in intensive care patients have examined the value of biomarkers to limit the duration of antibiotic therapy, and few have concentrated on its initiation. Although a recent meta-analysis suggests that PCT is helpful for differentiating sepsis from SIRS [63], the initiation of antibiotic therapy in ICU patients has been assessed in only two randomised open studies testing a PCT-based algorithm (Table 4) [33,34]. In the study by Layios et al. [34], there was no difference in the rate of initiation of therapy between the control group and the PCT-based group (where antibiotics were strongly discouraged if PCT was lower than 0.25 ng/mL, and strongly encouraged if PCT was higher than 1 ng/mL). In the multicentre study performed by Bouadma et al. [33] and using a similar algorithm, the risk reduction of initiating antibiotic therapy varied between 5% and 13% across centres. However, the small number of patients having CAP (n = 69) and the very low observance of the algorithm for withholding antibiotics when PCT levels were low (6%) in this study do not allow concluding on this point.

**Table 4:** Biomarkers and initiation or discontinuation of antibiotic therapy in adult ICU patients with sepsis

**Biomarker**	**Study* 1st author, year [ref]***	**Study design, patient selection (objective)**	**Nb of patients *n***	**Level of evidence**	**Primary endpoint and protocol**	**Main results PCT-guided vs. controls (ARR, absolute risk reduction)**
PCT	Layios N, [34]	Open, randomised controlled trial, 5 ICUs	509	High	Total antibiotic use in ICU patients when using a PCT-based algorithm for initiating antibiotics (lower PCT threshold for not initiating therapy: 0.25 ng/mL)	Percent days on antibiotics or overall DDD did not differ between the two groups. Withholding or withdrawing antibiotics similar overall (ARR = 3%) and with low PCT levels (PCT: 46.3%; controls: 32.7%; *p* = NS), or higher levels.
		Patients suspected of infection on admission or during the ICU stay (initiation of therapy)	PCT: 353			
			Ctr: 314			
PCT	Nobre V, [35]	Single-centre, open RCT;	79	Moderate	Total antibiotic days.	ARR antibiotic days: 3.5 (6 vs. 9.5 days; *p* = 0.15),
		PCT-guided withdrawing antibiotics vs. “standard care” (duration)ICU patients with severe sepsis/shock on admission or during ICU stay (excl. immunosuppressed patient or requiring prolonged therapy)	PCT: 39 (31 assessed)*		Recommend stopping antibiotics if PCT levels ≤ 90% of initial value but not before Day 3 (if baseline PCT level <1 ng/mL) or Day 5 (if baseline level ≥ 1 ng/mL).	Less overall ab exposure (504 vs. 655 ab days; *p* = 0.28); days alive without antibiotics at 28 days (15.3 vs. 13.3 days; *p* = 0.28). 28-d mortality: 20.5% vs. 20%
			Ctr: 40 (37 assessed)*			
			70% CA infections			*4 and 2 secondary exclusions for complicated infections (empyema, mastoiditis, abscess)
PCT	Bouadma L, [33]	Multicenter randomised open trial, 7 ICUs	630	High	Number of days alive and without antibiotics; noninferiority in terms of mortality by using a PCT-based algorithm for initiating or withdrawing antibiotics in those suspected of infection on admission or during the ICU stay (lower PCT threshold for not initiating or stopping therapy: 0.25 ng/mL)	ARR: 5% for initiating antibiotics (PCT: 91% vs. 96% in Ctr group).
		Sepsis in ICU patients, on admission or ICU-acquired (Initiation and duration)	PCT: 311			
			Ctr: 319			ARR for nb of antibiotic days: 2.7 days [1.4–4.1]
						Ab-free days by 28 d: 11.6 vs. 14.3 days
						28-d mortality : 21.2% vs. 20.4%; ARR = 0.8% [-4.6 to 6.2]
PCT	Stolz D, [69]	Multicentre open randomised trial, 7 ICUs (duration of therapy for VAP)	101	Moderate	Ab-free days alive at 28 days	Ab-free days at 28 d: 13 vs. 9.5 days
			PCT: 51		Discontinue ab if PCT <0.25 or <0.5 ng/ml and decrease by >80% from initial level	Ab duration: 10 vs. 15 days
			Ctr: 50			28-d mortality: 20% vs. 28%
PCT	Hochreiter M, [70]	Single-centre open randomised trial	110	Moderate	Reduction in ab duration	Mean Ab duration: 5.9 vs. 7.9 d
		Postoperative sepsis (duration)	PCT: 57		Discontinue ab if PCT <1.0 and clinical improvement, or sustained decrease to 25-35% initial value for 3 days	Mean ICU LOS:
			Ctr: 53			28-dMortality: 26.3% vs. 26.4%
PCT	Kopterides P, [71]	Meta-analysis of RCT in ICU patients (7 studies)	1131 patients	High	Various algorithms for discontinuation of Ab therapy	Duration ab : -2.1 [-2.5 to – 1.8] d
						Total Ab exposure: -4.2 [-5 to -3.4] days
						Ab free-days: 2.9 [1.9–3.9] days
						28-d mortality: OR = 0.93 [0.69-1.26]
**Summary table: Sepsis in ICU patients**						
	**Total number of patients, n**	**Highest level of evidence**	Directness*	Consistency**	**Overall strength of evidence**	**Number of studies, n**
	1010	High	Yes	Yes	**Initiation of therapy: low**	7
					**Discontinuation of therapy: high**	

*Directness: studies provide evidence of a direct association between a treatment or a given risk factor and a judgment criterion.

**Consistency: results from studies of similar level of evidence are not contradictory.

Relying on changes in PCT levels might be helpful for the initiation of antibiotic therapy in intensive care patients suspected of ICU-acquired infection; however, currently available data (on a total of 207 patients) are insufficient to base a recommendation on these [36,64]. One randomized, controlled study that enrolled 604 ICU patients has tested the diagnostic value of daily measurements of serum PCT levels (using a threshold of 1 ng/mL to rapidly initiate a diagnostic workup and protocolised therapy) [65]. The length of ICU stay and of mechanical ventilation were actually higher in the PCT arm (without difference in 28-day mortality), and time to adequate therapy was not lower (except for patients with bacteremia). Of note, antibiotic consumption was significantly higher in the PCT arm, as well as the total number of days spent in the ICU with three or more antibiotics.

In summary, we do not recommend using a threshold serum PCT value to help in the decision to initiate antibiotic therapy in ICU patients suspected of community-acquired pneumonia. There are insufficient data available to recommend using repeated PCT measurements and serum kinetics for the decision to initiate antibiotic therapy in ICU patients suspected of ICU-acquired infection.

### When can biomarkers help the decision to stop antibiotic therapy?

Given the number of studies examining this question and the high level of evidence generated, investigating this question was limited to examining randomized, controlled studies having tested a strategy based on biomarker measurement(s), to the exclusion of all other study designs. All studies in hospitalised patients used serum PCT level measurements, as there is no study testing the impact of using another biomarker in this specific indication; whereas several studies have tested the value of CRP for initiating antibiotics in pre-hospital care [24,66-68], none examined its potential impact on discontinuation of antibiotics, although several studies are ongoing (see http://www.clinicaltrials.gov/ct2/results?term=c+reactive+protein+and+duration&recr=&rslt=&type=&cond=&intr=&outc=&spons=&lead=&id=&state1=&cntry1=&state2=&cntry2=&state3=&cntry3=&locn=&gndr=&rcv_s=&rcv_e=&lup_s=&lup_e=). Accordingly, only studies using PCT levels are considered below.

### How can biomarkers be used to help decide on discontinuing antibiotic treatment?

To date, 14 trials have examined the clinical impact of PCT-guided antibiotic therapy and its discontinuation [15-20,22,23,33,35,69,70,72],[73]. Nine of these focused on the latter objective; four were conducted in prehospital care or emergency room, whereas the remaining five were conducted in ICU patients. Although specific stopping rules may vary across trials and population enrolled, all studies used a PCT-based algorithm to help decide on stopping antibiotics (Table 5).

**Table 5:** PCT-based algorithms used for discontinuing antibiotic therapy in randomized, clinical trials

**Author [ref], acronym**	**Setting**	**Population**	**Number of patients**	**Algorithm used**
Emergency department and ambulatory care				
Christ-Crain [18], ProCAP trial	Emergency room	CAP	302	PCT measured d4, d6, d8
			- 151 PCT-guided arm	Stopping antibiotics encouraged if PCT < 0.25μg/L; strongly encouraged if PCT < 0.1 μg/L
			- 151 control arm	If initial PCT >10μg/L, stop when decreased by ≥90%
Briel [15]	Ambulatory care	Lower RTI	458	PCT at d3
			- 232 PCT-guided arm	Encourage stopping if PCT d3 ≤ 0.25 μg/L
			- 226 control arm	
Schuetz [25]	Emergency room	Upper & lower RTI	1359	PCT at d3, d5, d7 if patient still hospitalised
			- 671 PCT-guided arm	Stop antibiotics when PCT ≤ 0.25 μg/L
				If initial PCT >10 μg/L, stop when decreased by ≥80%
			- 688 control arm	
Long W [22]	Emergency room	CAP	172	PCT at d1, d3, d6, & d8
			- 86 PCT-guided arm	Stop when PCT ≤ 0.25 μg/L
			- 86 control arm	
Intensive care unit				
Nobre [35]	ICU	Severe sepsis & septic shock	79	PCT d1 > 1 μg/l :
			- 39 PCT-guided arm	• Stop if PCT d5 decreased by > 90% or PCT < 0.25 μg/L
			- 40 control arm	PCT d1 < 1μg/l :
				• Stop if PCT d3 < 0.1 μg/l (but not before d5 if bacteremia)
Hochreiter [70]	ICU	Sepsis	110	Stop if clinical symptoms resolved and PCT < 1 μg/L (or dropped by 25% - 35% over 3 days if initial PCT > 1 μg/L)
		(Infection + 2 SIRS criteria)	- 57 PCT-guided arm	
			- 53 control arm	
Schroeder [73]	ICU	Severe sepsis after abdominal surgery	27	Stop if clinical symptoms resolved and PCT < 1 μg/L (or dropped by 25% - 35% over 3 days if initial PCT > 1 μg/L)
			- 14 PCT-guided arm	
			- 13 control arm	
Stolz [69], ProVAP trial	ICU	VAP	101	Daily PCT measurements PCT from d3 on
			- 51 PCT-guided arm	Stop when PCT < 0.5 μg/L or dropped by ≥ 80% from initial value but stopping discouraged if PCT >1 μg/L
			- 50 control arm	
Bouadma [33], PRORATA trial	ICU	Sepsis, severe sepsis	621	Daily PCT measurements from d3 on
			- 307 PCT-guided arm	Stop when PCT < 0.5 μg/L or dropped by ≥ 80% from initial value
			- 314 control arm	

*CAP* community-acquired pneumonia, *ICU* intensive care unit, *PCT* procalcitonin, *RTI* respiratory tract infection, *SIRS* systemic inflammatory response syndrome, *VAP* ventilator-associated pneumonia.

In outpatients and emergency room patients (excluding ICU patients), a serum PCT level below 0.25 ng/mL obtained 3 days or more after initiation of antibiotics, or a more than 80% decrease from the peak PCT level, allows stopping therapy.

Five studies have enrolled ICU patients with community- or hospital-acquired infection; four used a similar algorithm and the fifth used a different algorithm. It seems reasonable to recommend using the algorithm tested on the largest number of patients, i.e., as in the ProVAP et Prorata studies [33,69], where stopping therapy was strongly encouraged when the serum PCT level was <0.5 ng/mL at 3 days or more after initiating antibiotics, or an >80% decrease from the maximal serum PCT value was recorded.

### Does the site of infection (known, presumed, unknown) influence the utility of biomarkers to help withdrawing antibiotics?

In all studies examining the prognostic and follow-up value of PCT, the site of infection was known, to the exception of a few patients (n = 18) in the PRORATA study (10 and 8 in the PCT arm and control group, respectively). This small number does not allow any conclusion for this subgroup. It should be noted that patients having infective endocarditis, bone and joint infection, acute mediastinitis, intracerebral or intra-abdominal abscess were excluded from the above studies. Therefore, PCT-based algorithms cannot be used in these patients for discontinuing antibiotics.

Therefore, PCT-guided interruption of therapy can be used as indicated above in patients having a clinically documented site of infection to the exception of those sites listed above, which were excluded from clinical trials. When the site of infection is unknown, insufficient data are available to make a recommendation.

### Does microbiological documentation influences the clinical utility of biomarkers to help withdrawing antibiotics?

#### *Microbiologically documented infection*

In the PRORATA study, most patients enrolled (70%) had microbiologically documented infection (222 and 213 in the PCT and control group, respectively) [33]. In the subgroup of 108 patients having positive blood cultures (55 and 53 in the PCT and control group, respectively), those randomised to the PCT-guided algorithm received 3 days less antibiotics than those enrolled in the control group (IC95%, -6 to 0.1 day, *p* = 0.06), without difference in mortality rate.

In the ProHosp trial, 72 patients had positive blood cultures [25]; patients enrolled in the PCT-guided therapy group received 5 days less antibiotics (10.3 vs. 15.1 days). Among 237 patients with bacteremic LRTI included in a recent meta-analysis [32], those treated with the aid of a PCT-based algorithm had 3.5 less antibiotic days (95% CI 1.55-5.54, *p* < 0.001), without significant difference in mortality rate (OR 1.09; 95% CI 0.51-2.31).

#### *Lack of microbiological documentation*

Most studies conducted outside of the ICU have enrolled patients in whom microbiological documentation was lacking. Although this specific subgroup has not been examined separately in individual studies or meta-analyses, it seems reasonable to recommend using a PCT algorithm in the non-ICU population to help decide stopping antibiotic therapy.

Most ICU patients enrolled in the above mentioned studies had documented infection. However, in the PRORATA study [33], 186 episodes were nondocumented and those treated in the PCT-guided therapy arm had a nonsignificant reduction in antibiotic days (2.4 less days), with no difference in mortality rate. Therefore, the documentation of infection does not appear to influence the impact of PCT-guided withdrawal of therapy, whether in ICU or non-ICU populations.

### Does an immunocompromised status of patients influence the use of biomarkers for stopping antibiotic therapy?

Among the nine trials testing the impact of PCT-guided discontinuation of therapy, only the PRORATA trial [33] enrolled immunocompromised patients in the ICU. This trial enrolled patients having HIV infection or AIDS, organ transplant recipients, patients having haematological malignancy or receiving chemotherapy or radiation therapy, immunosuppressive agents or long-term steroids, to the notable exception of bone marrow transplant recipients or those having severe neutropenia (<500 leucocytes/mm^3^). About a hundred such immunocompromised patients were included (47 and 51 in the PCT-guided therapy group and control group, respectively). In this subgroup, PCT-guided discontinuation of therapy was associated with a significantly reduced duration of therapy (3.6 days; 95% CI, 0.2-7 days), without apparent effect on morbidity or mortality (control vs. PCT, -7.1%; 95% CI -18.7 to 4.5%).

Therefore, PCT-guided algorithms to reduce the duration of antibiotic therapy can be used safely in immunocompromised patients, to the exception of neutropenic patients (<500 neutrophils/mm^3^) or bone marrow transplant recipients, which were excluded from trials and in whom PCT-guided therapy cannot be recommended.

### Does the impact of PCT-guided therapy vary according to the severity of acute illness?

#### *Systematic reviews and meta-analyses*

Six studies were reviewed in the meta-analysis by Tang et al. [74], totalling 1,548 patients. Four of these trials enrolled patients with suspected LRTI, two studies enrolled patients with sepsis, and one focused on severe infections in surgical ICU patients. Algorithms used varied across studies, using 2, 3, or 4 PCT levels for decision-making. There was a nonsignificant trend to a reduced duration of antibiotic therapy among LRTI studies (*p* = 0.067), which showed significant heterogeneity between trials. Conversely, in the other three studies of sepsis and surgical patients that enrolled more severe patients, no substantial heterogeneity was observed, and PCT-based algorithm for discontinuation of antibiotics were associated with a significant reduction in antibiotic duration, without apparent deleterious effect. This meta-analysis did not, however, stratify trials according to the severity of illness. In the subgroup of four studies having the strongest design (including 3 of the 4 studies on LRTI), there was a significant reduction in the duration of antibiotic therapy, but a significant heterogeneity persisted.

Significant heterogeneity also was evidenced in a recent meta-analysis including eight studies of LRTI [75]. There was one trial in patients with acute exacerbation of COPD, one on patients with CAP, two on patients with upper and lower RTI [16], and three on LRTI. A significant reduction of antibiotic duration was noted in all but one study [16], where a PCT-based algorithm was not used. Similarly to the previous analysis, this meta-analysis did not stratify patients according to their severity.

In the more recent systematic review by Schuetz et al. focusing on LRTI [32], 14 trials totalling 4,221 patients were analysed. A reduced rate of treatment initiation was confirmed in studies performed in primary care and patients having upper or lower RTI or acute bronchitis. Trials performed in the emergency department or the ICU and enrolling patients with LRTI, whether CAP or VAP, also found a reduction in the duration of antibiotic therapy. A sensitivity analysis showed no significant difference in the reduction of antibiotic duration according to the type of LRTI and site of care. It was however noted that the observance of clinical algorithms was lower in the ICU setting than at other sites.

#### *Individual studies*

Christ-Crain et al. [18] reported that PCT levels increased with the severity of illness, as assessed by the pneumonia severity index (PSI). However, the duration of antibiotic prescription decreased similarly with PCT-guided therapy in the low-risk (PSI I-III) or high-risk (PSI IV-V) group. In another study in patients with LRTI from the same group [17], only admission PCT levels were recorded but not duration of therapy. In the PROHOSP study [25], the reduction of antibiotic duration with PCT-guided therapy was more marked among patients having acute bronchitis (-65%) than among patients with acute exacerbation of COPD (-50%), and lowest (-32%), but still strongly significant, among those with CAP.

In the trial conducted by Stolz et al. in patients with acute exacerbation of COPD [20], the impact of PCT-guided therapy was not analysed according to the severity of the episode or of the underlying COPD, which included all severity stages.

Briel et al. enrolled patients with RTI from various causes, including upper respiratory tract infection or acute bronchitis, CAP, or acute exacerbation of COPD [15]. The reduction in antibiotic duration was more marked in the former group than in those with CAP or acute exacerbation of COPD.

The proREAL trial also enrolled patients (n = 1,759) with acute bronchitis, exacerbation of COPD, and CAP [27]. The observance of the algorithm was 81%, 70%, and 64% respectively, confirming other observations [32,33] that the observance decreases with increasing severity of illness. Of note, the algorithm used in that study included both the clinical context and PCT levels (Table 3).

Long et al. also found a reduction in antibiotic duration of patients with CAP [22]. However, this study enrolled only patients with nonsevere pneumonia and cannot inform this assessment according to severity of illness. In trials dealing with the more severe infections (VAP, sepsis) [33,35,65,66], analyses have not been stratified according to the level of severity.

## Summary and conclusions

In view of currently available data, PCT is the only biomarker that has been extensively studied so far to help decision-making in discontinuing antibiotic therapy in adults. In clinical practice, an algorithm should be used, based on PCT levels on day 1 (reference value), then at day 2–3, and every 48 h until antibiotic therapy is stopped.

In nonimmunocompromised patients treated for RTI as outpatients or hospitalised in regular wards, the following stopping rule can be used: discontinuation of antibiotic therapy if the PCT level at day 3 is lower than 0.25 ng/mL or has decreased by >80-90% relative to the maximal value initially recorded, whether or not microbiological documentation has been obtained.

For patients hospitalised in ICU, including immunocompromised patients (but not neutropenic patients or bone marrow transplant recipients), the following decision rule can be suggested for nonbacteremic patients with a known site of infection (whether or not microbiological documentation is obtained): stopping antibiotics if the PCT level at day 3 is <0.5 ng/ml or has decreased by >80% relative to the highest level recorded during this episode. In bacteremic patients, a minimal duration of therapy of 5 days is recommended.

Overall, the severity of the infectious episode does not appear to alter substantially the impact of PCT measurements on the reduction of antibiotic duration; however, the magnitude of the reduction is more marked in infections of lesser severity, which likely reflects at least two factors: 1) the less common indications for antibiotic therapy in such conditions, which is in contrast to the high tendency among physicians to initiate therapy when in doubt on the aetiology; 2) the better observance of decision algorithms by physicians, likely related to their greater confidence in the lack of serious risk associated with withholding or withdrawing antibiotics in these low-severity patients.

## Abbreviations

AIDS: Acquired immunodeficiency syndrome; AUC: Area under the curve; AUROC: Area under the receiver operating curve; BM: Bacterial meningitis; BMS: Bacterial meningitis score; CAP: Community-acquired pneumonia; COPD: Chronic obstructive pulmonary disease; CRP: C-Reactive protein; CSF: Cerebrospinal fluid; HIV: Human immunodeficiency virus; ICU: Intensive care unit; IFN-Υ: Interferon-gamma; IL: Interleukin; LRTI: Lower respiratory tract infection; PCT: Procalcitonin; PMN: Polymorphonuclear neutrophil; PPV: Positive predictive value; PSI: Pneumonia severity index; ROC: Receiver Operating Characteristic curve; RTI: Respiratory tract infection; SIRS: systemic inflammatory response syndrome; sTREM-1: soluble Triggering Receptor Expressed on Myeloid cells-1; TNF: Tumor necrosis factor; VAP: ventilator-associated pneumonia.

## Competing interests

Meetings of the expert panel group were supported in part by an unrestricted grant from Thermo-Fisher to the Maurice Rapin Institute. This company had no role in the work of the panel, or in the drafting, revision or final approval of the manuscript. A-MD declared participation as an investigator to the UTAPE study, sponsored by Thermo-Fisher, and her institution received funding for analytic studies of CT-pro AVP using Kryptor. CB-B was an investigator in the Prorata trial. MC holds a patent for the MENINGITEST in Europe (European patent EP1977244), USA and Canada, and has a patent pending for the REFLUTEST (WO2010/109089). C-EL was an investigator for the Prorata trial, and received lectures honoraria from Thermo-Fisher and Biomérieux. NR is coordinating the UTAPE study on biomarkers in COPD exacerbations seen in the emergency department, sponsored by Thermo-Fischer. Y-EC received research funding from Thermo-Fisher, Roche Diagnosis and Nephrotek. P-EC received speaking fees from Thermo-Fisher. CG-LG received funding by Thermo-Fisher for attending a scientific meeting. JP received speaking fees for giving lectures within symposia organized by ThermoFischer. J-PB, RG, SL, BM, J-PQ, FP, YP, and J-PS have declared no competing interest in relation to the subject of this manuscript.

## Authors' contributions

All panel members contributed to the panel discussions and analyses. Each panel members contributed to drafting different sections of the manuscript: A-MD, YP, FP, SR, and BM drafted part I; J-PQ, SL, Y-EC, J-PS, CG-L, MC, and RG drafted part II; and C-EL, NR, J-PB, JP, and CB-B drafted part III. A-MD, J-PQ, C-EL, RG, BM, MC, and CB-B extensively reviewed the consolidated manuscript and all authors approved its final version.
